# Analysis of respiratory virus detection in hospitalized children with acute respiratory infection during the COVID-19 pandemic

**DOI:** 10.1186/s12985-023-02218-5

**Published:** 2023-11-02

**Authors:** Ruoya Wu, Jianwei Zhang, Liyan Mo

**Affiliations:** 1 Nursing department, Shaoxing Maternity and Child Health Care Hospital, 305 East Street, Yuecheng District, Shaoxing, Zhejiang P. R. China; 2 Department of Pediatrics, Shaoxing Maternity and Child Health Care Hospital, 305 East Street, Yuecheng District, Shaoxing, Zhejiang P. R. China

**Keywords:** COVID-19 pandemic, Acute Respiratory Infections, Respiratory virus, Children

## Abstract

**Objective:**

It is now understood that the Coronavirus disease 2019 (COVID-19) pandemic and its associated containment measures have influenced the epidemiology of other respiratory viruses. This study aimed to characterize respiratory virus infections in pediatric patients hospitalized for acute respiratory infections (ARIs) in East China both prior to and during the COVID-19 pandemic.

**Methods:**

We collected nasal secretions from 9782 pediatric ARI patients admitted to Shaoxing Maternal and Child Health Care Hospital between January 2018 and December 2022. We analyzed and compared changes in viral detection rates, epidemiological features, and clinical characteristics.

**Results:**

A total of 1633 strains from 7 common respiratory viruses were identified, with an overall positive rate of 16.35% (n = 821/5021) in 2018–2019 and 17.06% (n = 812/4761) in 2020–2022. Compared to 2018–2019, the positive rate for RSV significantly increased in 2020–2022, while detection rates for ADV, PIV-2, PIV-3, and flu-B showed reductions (P < 0.05). The RSV-positive rate experienced a more significant increase in winter compared to other seasons both before and during COVID-19 (P < 0.05), whereas PIV-3 predominantly circulated in spring and summer before COVID-19.

**Conclusion:**

During the COVID-19 pandemic, marked variations in age distribution and seasonality of respiratory virus infections were observed among hospitalized children with ARIs in East China. Non-pharmaceutical interventions (NPIs) implemented during the pandemic yielded a limited impact on common respiratory viruses.

**Supplementary Information:**

The online version contains supplementary material available at 10.1186/s12985-023-02218-5.

## Introduction

Acute respiratory infections (ARIs) tend to occur predominantly in children and have a significant impact on their health [1], with over 80% of respiratory tract infections in children attributed to viruses [2], including adenovirus (ADV), respiratory syncytial virus (RSV), influenza virus (Flu) types A and B, and parainfluenza virus (PIV) types 1, 2, and 3 [3–5]. Respiratory viruses exhibit distinct epidemic patterns and are susceptible to influences from environmental, climatic, and human mobility factors [6, 7]. Amidst epidemic respiratory viruses, non-pharmaceutical interventions (NPIs) frequently constitute the initial line of defense to slow down and mitigate their spread within the population [8].

Upon the declaration of coronavirus disease 2019 (COVID-19) as a pandemic in January 2020, a comprehensive set of public health preventive NPI measures was implemented to curb disease transmission, encompassing social distancing, school closures, mask mandates, travel restrictions, enhancements in personal hygiene, and border closures in China. Consequently, a marked reduction in the prevalence of other commonplace respiratory viruses occurred across most temperate regions [9, 10]. To comprehensively examine the impact of COVID-19 and the associated preventive measures on the epidemiology of prevalent respiratory viruses, especially RSV, we conducted a retrospective analysis of viral pathogen epidemiological characteristics in clinical samples collected from children with ARIs in East China. This analysis encompassed the period from January 2018 to December 2019, juxtaposed with data from January 2020 to December 2022. Based on these findings, we formulated preventive strategies against viral infections in children.

## Methods

### Study design and participants

A retrospective survey-based investigation was conducted at a tertiary maternal and child health facility, the Shaoxing Maternal and Child Health Care Hospital, in East China, from January 2018 to December 2022. A total of 9782 pediatric patients aged below 14 years diagnosed with acute respiratory infections (ARIs) were included. ARIs diagnosis was established by skilled medical practitioners, encompassing both upper and lower respiratory tract infections. The study encompassed screening for numerous respiratory pathogens. Inclusion criteria comprised: (1) presence of fever or an ear temperature measurement ≥ 37.5 °C; (2) manifestation of one or more respiratory symptoms within a 14-day period (cough, sore throat, sputum, shortness of breath, abnormal lung auscultation findings such as rales or wheezes, tachypnea, and chest pain); and (3) cases necessitating hospitalization [11, 12]. Exclusion criteria were set for children: (1) with more than one visit within a week; (2) experiencing a hospital-acquired infection; (3) possessing congenital pulmonary airway malformation or an impaired immune system; and (4) being infected with COVID-19. Given the retrospective nature of our study, the utilization of electronic hospital records, and the absence of additional physical risks to participants, the Medical Ethics Committee of our institution waived the requirement for informed consent.

### Sample collection and laboratory test

Respiratory samples acquired via nasal swabs were collected from all eligible ARI patients within the initial 24 h of hospitalization, both prior to and during the COVID-19 period. A nurse conducted nasal swabs collection and transferred the swabs into a conical tube containing 2 mL of sterile saline. These samples were promptly dispatched to the Department of Laboratory Medicine within the hospital for analysis. Numerous respiratory pathogens were detected by direct immunofluorescence technique.

The collected clinical samples underwent a series of steps: vortexing for 30 s, centrifugation at 1000×g for 10 min to yield approximately 100µL supernatant, and preparation of a cell suspension through repeated pipetting. Subsequently, the cell suspension was applied onto an 8-well chamber slide, and 25 µl of the corresponding fluorescent antibody was added dropwise. One well was reserved as a negative control. Following incubation at 37.0℃ for 30 min, a drop of blocking solution was introduced after a washing step, and examination under a fluorescence microscope was conducted following slide sealing. The FITC-labeled virus-specific monoclonal antibody interacted with the relevant viral antigen in cells, forming an antigen-antibody complex, which emitted apple green fluorescence, while the negative regions were stained red using Evans blue. A positive outcome was established when at least two green fluorescent cells were observed within each field of vision under the microscope at a magnification of 200 times. The respiratory tract virus antigen test was supplied by Shanghai Haide Diagnostic Co., LTD (lot number: 186,325), encompassing the detection of RSV, ADV, Flu A and B, and PIV1, 2, and 3.

### Statistical analysis

Database establishment and analysis were performed using SPSS19.0 software. Categorical variables were reported as frequencies and percentages. Group comparisons were accomplished using the chi-square test or Fisher exact probability method. A p-value < 0.05 was statistically significant.

## Results

### General clinical features

Among the cohort of 9782 children diagnosed with ARIs, 5021 cases (51.3%) were identified prior to the onset of the COVID-19 pandemic (2018–2019), while 4761 cases (48.7%) were diagnosed during the pandemic period (2020–2022). Compared with the pre-COVID, the proportion of children aged 0 ~ < 1 years on ARIs was lower during the COVID-19 pandemic (p < 0.05). Gender distribution exhibited no significant variation between the two periods (*p* > 0.05), as indicated in Table 1.

**Table 1:** Epidemiologic characteristics of respiratory virus detection among hospitalized children with ARIs from 2018 to 2022

	2018–2019	2020–2022	χ^2^ value	P value
	(n = 5021)	(n = 4761)		
**Characteristics, n(%)**				
Age				
0~<1y	2688 (53.53)	2311 (48.54)	24.400	0.000
1~<3y	1223 (24.36)	1256 (26.38)	5.288	0.021
3~<7y	989 (19.70)	1036 (21.76)	6.335	0.012
7~<14y	121 (2.41)	158 (3.32)	7.284	0.007
Gender				
Male	2948 (58.70)	2799 (58.8)	0.006	0.939
Female	2073 (41.30)	1962 (41.2)		
**Detection of viruses, n(%)**				
ADV	36 (0.72)	14 (0.29)	8.678	0.003
RSV	471 (9.38)	621 (13.04)	33.061	0.000
Flu A	56 (1.12)	54 (1.13)	0.008	0.929
Flu B	35 (0.70)	13 (0.27)	8.998	0.003
PIV-1	33 (0.66)	22 (0.46)	1.665	0.197
PIV-2	31 (0.62)	5 (0.11)	21.674	0.000
PIV-3	159 (3.17)	83 (1.74)	20.520	0.000
Total	821 (16.35)	812 (17.06)	0.871	0.035
**Mixed virus-positive specimens**				
ADV + PIV-2	1 (0.02)	0 (0.00)	0.948	0.330
ADV + PIV-3	1 (0.02)	0 (0.00)	0.948	0.330
RSV + PIV-1	1 (0.02)	1 (0.02)	0.001	0.970
RSV + PIV-3	1 (0.02)	4 (0.08)	1.965	0.161
PIV-2 + PIV-3	1 (0.02)	0 (0.00)	0.948	0.330
PIV-1 + PIV-3	1 (0.02)	0 (0.00)	0.948	0.330
Total	6 (0.12)	5 (0.10)	0.046	0.831
**Total**	827 (16.47)	817 (17.16)	0.831	0.362

Between 2018 and 2022, the yearly counts of ARIs cases were 2356, 2665, 1092, 1681, and 1988, with the lowest number of inpatient ARIs cases in 2020. The annual percentage of positive cases was 12.99%, 19.32%, 20.32%, 19.45%, and 12.68%, as presented in Table S1. Indeed, the Chinese government implemented prompt and stringent epidemic prevention measures starting in January 2020, including home isolation and mask mandates. From December 2021, children above the age of 3 (except those with specific contraindications) received SARS-CoV-2 vaccinations in Shaoxing. The positive rate of respiratory viruses among children over 3 years old was 8.31% (52/626) in 2022 and 7.61% (29/381) in 2021. Importantly, the detection rate of common respiratory viruses did not exhibit a significant decrease after the introduction of the new coronavirus vaccination (*P = 0.69*).

### Overall detection of respiratory viruses

**Fig. 1:**
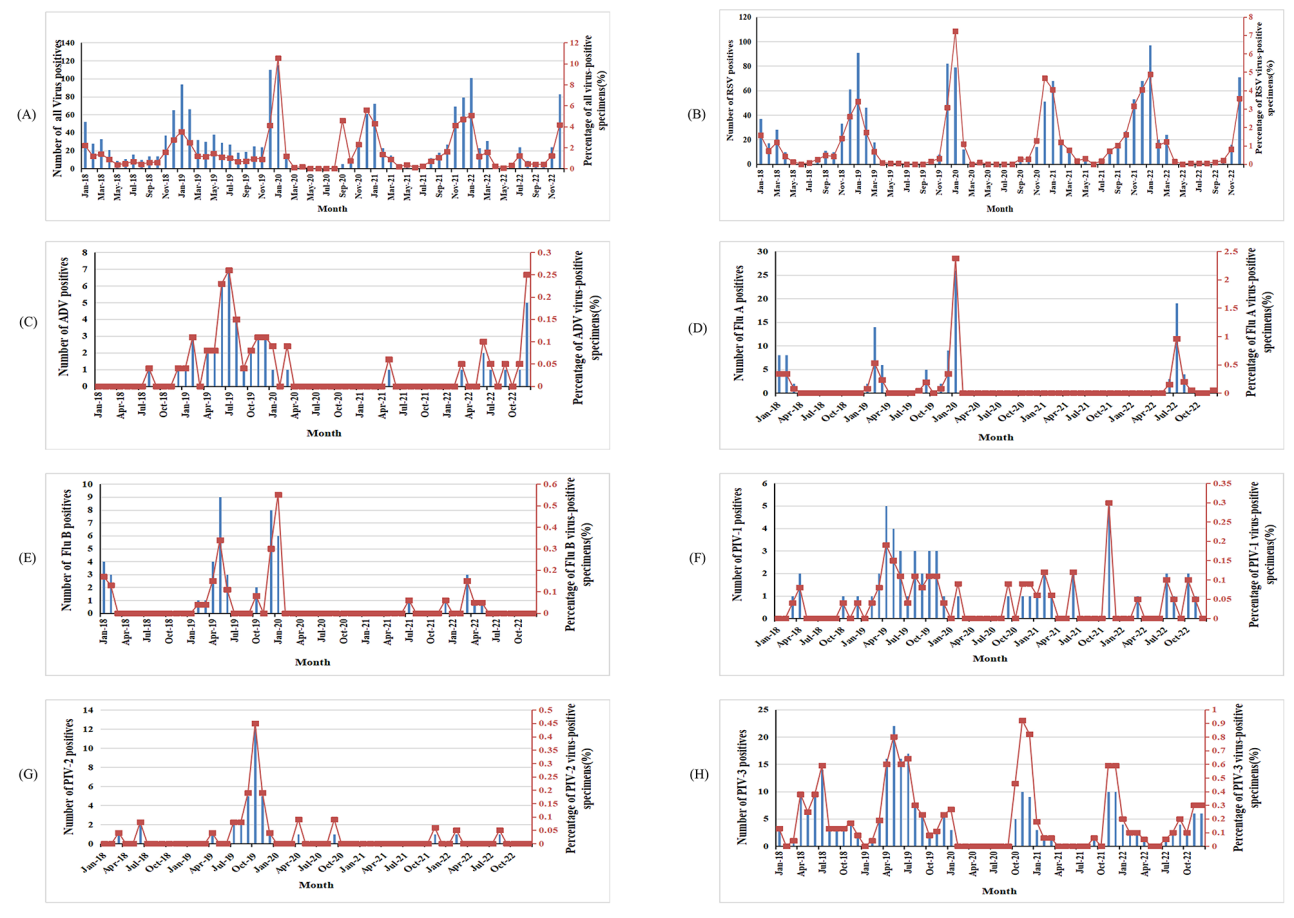
Positive cases and infection rates of respiratory viruses among hospitalized children with ARIs from 2018 to 2022. **A**: Total positive cases and infection rates for all respiratory viruses. **B**: Positive cases and infection rates of RSV. **C**: Positive cases and infection rates of ADV. **D**: Positive cases and infection rates of Flu (A) **E**: Positive cases and infection rates of Flu (B) **F**: Positive cases and infection rates of PIV-1. **G**: Positive cases and infection rates of PIV-2. **H**: Positive cases and infection rates of PIV-3.

The identification of one or more viruses was observed in 16.35% (n = 821/5021) of samples prior to COVID-19 and 17.06% (n = 812/4761) during the pandemic, as detailed in Table 1. The cumulative detection rate of respiratory viruses experienced a decline from January to April and increased from October to December during both study periods (Fig. 1A).

RSV emerged as the predominant virus prior to and during COVID-19, accounting for 50.79% of positive samples in 2018–2019 and 76.48% in 2020–2022. Notably, the RSV positive rate during 2020–2022 exhibited a more significant increase than in 2018–2019 (13.04% vs. 9.38%, *p* < 0.05). Conversely, the positive rates of ADV, PIV2 and 3, and Flu B experienced more significant reductions during 2020–2022 compared to 2018–2019 (*p* < 0.05), while the positive rates for Flu A and PIV1 exhibited no significant differences between the two periods (*p* > 0.05) (Table 1).

The temporal distribution of infections and positive rates is depicted in Fig. 1B, wherein RSV infections and positive rates displayed seasonal fluctuations annually between September and May from 2018 to 2019, 2020 to 2021, and 2021 to 2022. A notable surge occurred from December 2019 to January 2020. ADV infections and positive rates exhibited seasonal fluctuations between January 2019 and January 2020, with a sharp decline between April 2020 and January 2022. Flu A infections and positive rates remained low between April 2020 and April 2022, with a modest increase observed in children during the COVID-19 recovery phase from May 2022 to September 2022. Flu B infections and positive rates were low between April 2020 and April 2021, with a subsequent modest increase after July 2021, although remaining lower compared to the same period prior to the COVID-19 outbreak. Notably, PIV1, PIV2, and PIV3 infections declined after January 2020 (Fig. 1C H).

A total of 11 samples yielded positive results for two viruses, with 6 instances prior to COVID-19 and 5 during the pandemic (0.12% vs. 0.10%, *p* > 0.05). Throughout COVID-19, the most prevalent mixed infection was ADV in conjunction with PIV3, accounting for 25.71% of mixed infection cases. Nevertheless, cases of more than two concurrent virus detections were not observed during the two study periods.

### Comparison of the age distribution of respiratory viruses

Patients were classified into four age groups. Table 2 illustrates the detection rates of respiratory viruses in different age groups. Prior to and during COVID-19, the overall positive rate peaked at 19.94% and 23.15%, respectively, within the 1–12 month age group. These rates exhibited a descending trend with increasing age of the enrolled children. Compared to the period before COVID-19, the total positive rates within the 0–12 month age group were significantly higher during the pandemic (*p* < 0.05), while significant differences were not noted in other age groups (Table S1).

**Table 2:** Age distribution comparison of respiratory virus infection in hospitalized children with ARIs between 2018–2019 and 2020–2022

Year	Age Group (Y)	Total number of cases	Percentage of virus-positive specimens [n(%)]							Total positive cases
			ADV	RSV	Flu A	Flu B	PIV-1	PIV-2	PIV-3	
2018–2019	infant (0~<1)	2688	4(0.15)	352(13.10)	29(1.08)	12(0.45)	17(0.63)	19(0.71)	103(3.83)	536(19.94)
	child (1~<3)	1223	10(0.82)	76(6.21)	19(1.55)	7(0.57)	10(0.82)	5(0.41)	46(3.76)	173(14.15)
	preschool (3~<7)	989	22(2.22)	41(4.15)	7(0.71)	13(1.31)	6(0.61)	6(0.61)	10(1.01)	105(10.62)
	school age (7~<14)	121	0(0.00)	2(1.65)	1(0.83)	3(2.48)	0(0.00)	1(0.83)	0(0.00)	7(5.79)
	χ^2^		38.241	98.442	3.486	12.087	0.654	1.622	29.137	63.322
	P		0.000	0.000	0.286	0.006	0.848	0.585	0.000	0.000
Year	Age Group(Y)	Total number of cases	Percentage of virus-positive specimens [n(%)]							Total positive cases
			ADV	RSV	Flu A	Flu B	PIV-1	PIV-2	PIV-3	
2020–2022	infant (0~<1)	2311	2(0.09)	443(19.17)	13(0.56)	8(0.35)	9(0.39)	4(0.17)	56(2.42)	535(23.15)
	child (1~<3)	1256	5(0.40)	119(9.47)	19(1.51)	3(0.24)	11(0.88)	0(0.00)	16(1.27)	173(13.77)
	preschool (3~<7)	1036	7(0.68)	58(5.60)	20(1.93)	1(0.10)	2(0.19)	0(0.00)	11(1.06)	99(9.56)
	school age (7~<14)	158	0(0.00)	1(0.63)	2(1.27)	1(0.63)	0(0.00)	1(0.63)	0(0.00)	5(3.16)
	χ^2^		9.470	162.647	14.223	2.445	7.303	7.627	13.465	77.955
	P		0.024	0.000	0.003	0.458	0.063	0.054	0.004	0.000

The dominant viruses varied across the different age groups. Before and during COVID-19, although all seven viruses were not detected in each age group, RSV remained the most prevalent among those under 7 years. In 2018–2019, PIV-3 was the second most dominant virus in the < 3 years age group, while ADV held this position in the 3–7 years age group. In 2020–2022, PIV-3 was the second most dominant virus in the < 1 year age group, while Flu A took this place in the 1–7 years age group. Notably, the RSV detection rate among different age groups was higher in 2020–2022 than in 2018–2019, especially among infants (*p* < 0.001).

### The seasonal distribution of respiratory viruses

Table 3 provides an overview of the positive sample detection for each virus across different months before and during COVID-19. In general, the prevalence of respiratory viruses demonstrated higher rates during winter compared to other seasons, with total positive rates of 30.90% and 33.24% before COVID-19 and during the pandemic, respectively. In contrast to the equivalent period preceding COVID-19, the total positive rates during winter in the COVID-19 era exhibited no significant difference (*p* > 0.05). During 2020–2022, the total positive rates during spring and summer seasons significantly decreased (*p* < 0.05), increased significantly in autumn (*p* < 0.05).

**Table 3:** Comparison of seasonal distribution of respiratory virus infection among hospitalized children with ARIs between 2018–2019 and 2020–2022

Time	Total number of cases	Percentage of virus-positive specimens [n(%)]							Total positive cases
		ADV	RSV	Flu A	Flu B	PIV-1	PIV-2	PIV-3	
Winter in 2018–2019 year(12 m ~ 2 m)	1343	8(0.60)	334(24.87)	41(3.05)	16(1.19)	3(0.22)	1(0.07)	12(0.89)	415(30.90)
Winter in 2020–2022 year(12 m ~ 2 m)	1501	6(0.40)	415(27.65)	27(1.80)	7(0.47)	5(0.33)	1(0.07)	38(2.53)	499(33.24)
χ^2^		0.556	2.821	4.776	4.645	0.304	0.006	11.012	1.785
P		0.456	0.093	0.029	0.031	0.581	0.937	0.001	0.182
Time	Total number of cases	Percentage of virus-positive specimens [n(%)]							Total positive cases
		ADV	RSV	Flu A	Flu B	PIV-1	PIV-2	PIV-3	
Spring in 2018–2019 year(3 m ~ 5 m)	1254	4(0.32)	62(4.94)	7(0.56)	14(1.12)	14(1.12)	2(0.16)	59(4.70)	162(12.92)
Spring in 2020–2022 year(3 m ~ 5 m)	997	3(0.30)	49(4.91)	0(0.00)	5(0.50)	2(0.20)	1(0.10)	4(0.40)	64(6.42)
χ^2^		0.006	0.001	5.583	2.509	6.601	0.146	37.816	25.977
P		0.939	0.974	0.018	0.113	0.010	0.702	0.000	0.000
Time	Total number of cases	Percentage of virus-positive specimens [n(%)]							Total positive cases
		ADV	RSV	Flu A	Flu B	PIV-1	PIV-2	PIV-3	
Summer in 2018–2019 year (6 m ~ 8 m)	1163	18(1.55)	9(0.77)	1(0.09)	3(0.26)	7(0.60)	6(0.52)	67(5.76)	111(9.54)
Summer in 2020–2022 year(6 m ~ 8 m)	998	3(0.30)	18(1.80)	26(2.61)	2(0.20)	5(0.50)	1(0.10)	3(0.30)	58(5.81)
X^2^		8.681	4.616	27.627	0.077	0.099	2.875	51.092	10.381
P		0.003	0.032	0.000	0.781	0.753	0.090	0.000	0.001
Time	Total number of cases	Percentage of virus-positive specimens [n(%)]							Total positive cases
		ADV	RSV	Flu A	Flu B	PIV-1	PIV-2	PIV-3	
Autumn in 2018–2019 year(9 m ~ 11 m)	1261	6(0.48)	66(5.23)	7(0.56)	2(0.16)	9(0.71)	22(1.74)	21(1.67)	133(10.55)
Autumn in 2020–2022 year(9 m ~ 11 m)	1265	2(0.16)	139(10.99)	1(0.08)	0(0.00)	10(0.79)	2(0.16)	38(3.00)	192(15.18)
X^2^		2.019	28.040	4.533	2.008	0.050	16.891	4.960	12.079
P		0.155	0.000	0.033	0.156	0.823	0.000	0.026	0.001

**Fig. 2:**
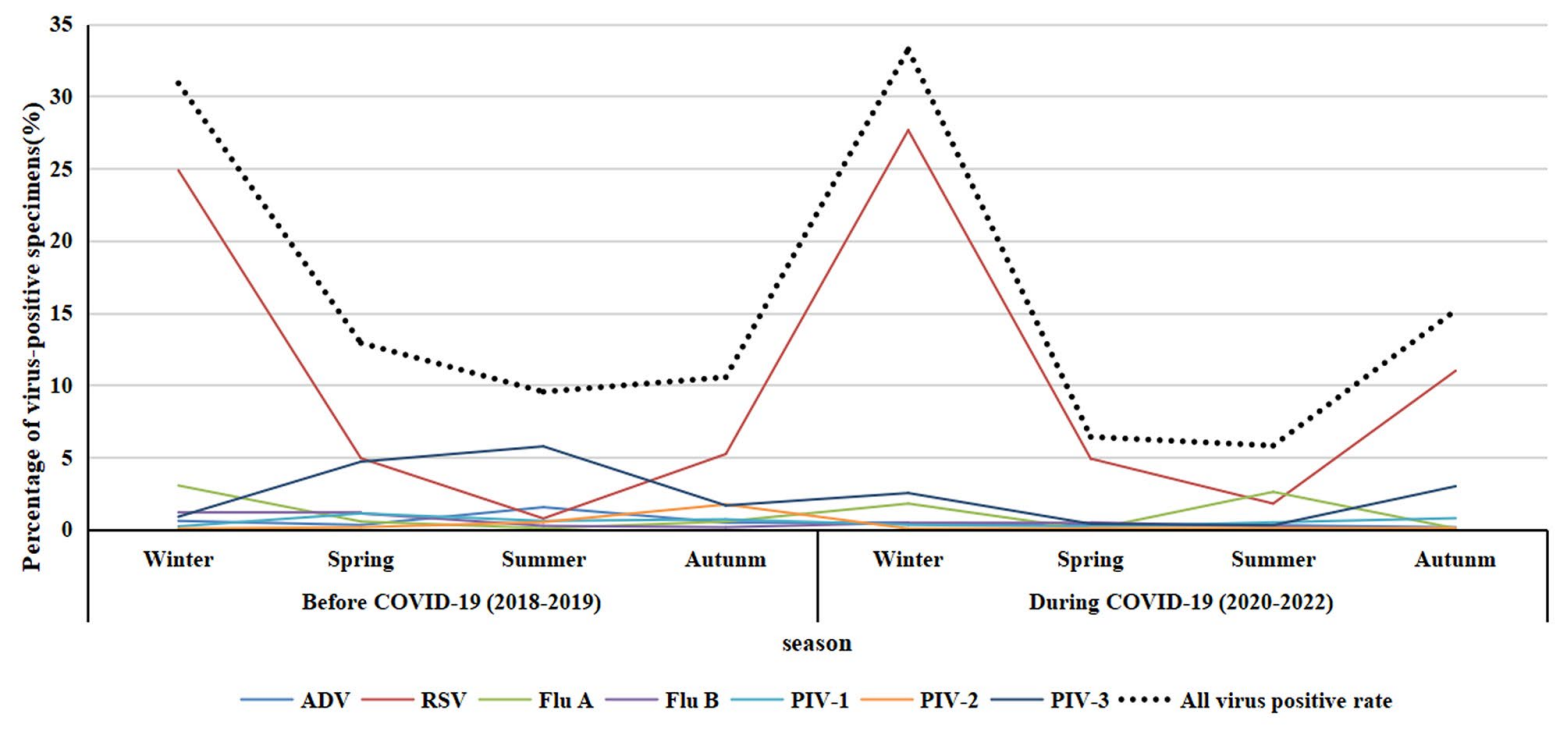
Seasonal distribution trends of respiratory virus infection in hospitalized children with acute respiratory infection, 2018–2019 and 2020–2022

Figure 2 further illustrates that both before and during COVID-19, the RSV-positive rate during winter was notably higher than in other seasons (*p* < 0.05). During COVID-19, Flu A exhibited a higher positive rate (2.61%) in summer, contrasting with the higher rate (3.05%) in winter before the pandemic. Conversely, the PIV3 positive rate during the summer of the pandemic (0.3%) was lower than that during the same period before COVID-19 (5.76%).

## Discussion

In January 2020, Zhejiang province escalated its emergency response to Level 1 in response to the COVID-19 pandemic. Several non-pharmaceutical interventions were implemented to mitigate virus spread, including mask-wearing, frequent hand washing, social distancing, and indoor ventilation. During the pandemic, children in Shaoxing were required to stay home and participate in online learning until schools reopened. Furthermore, from December 2021, all children over 3 years old (excluding those with contraindications) were administered SARS-CoV-2 vaccinations. These measures effectively slowed and controlled the spread of COVID-19 in Shaoxing until their relaxation in December 2022. It is noteworthy that prior to COVID-19, such rigorous measures and large-scale lockdowns had not been implemented. Respiratory virus infections in children have always garnered attention globally, with considerable efforts to reduce the prevalence of common childhood respiratory viruses alongside COVID-19 containment [2, 13, 14]. Remarkably, virus-positive samples increased by 7.2% compared to the pre-COVID-19 period, while detection percentages remained relatively unaffected. In contrast to the literature, we found that NPIs during the pandemic had a minimal impact on the circulation of common respiratory viruses [2]. This increase in cases may be attributed to a combination of factors, including a potential decline in population immunity and a relaxation of COVID-19 restrictions.

Amidst the COVID-19 outbreak, the test positivity rate witnessed a significant increase, primarily attributed to RSV, which exhibited a substantial surge consistent with findings reported in Australia [14, 15] and New Zealand. This surge might correspond to the relaxation of non-pharmaceutical interventions, extensive respiratory virus testing to rule out COVID-19, and possible changes in the environmental resistance of RSV strains [16–18], underscoring the dual nature of NPIs. This underscores the importance of recognizing NPIs as a two-edged sword, emphasizing the critical need for active and continuous epidemiological surveillance and timely adaptation of immunization strategies. The epidemiological pattern of RSV infection among children underwent significant shifts before and after the advent of COVID-19 in East China. Furthermore, a marked reduction in ADV, Flu B, and PIV-2 and − 3 during the COVID-19 period aligns with findings from other studies [19, 20].

In the present study, infants aged 1–12 months were notably susceptible to respiratory viruses (especially RSV) both pre-COVID-19 and during the pandemic. Interestingly, the prevalence of respiratory infections in this age group was nearly higher after COVID-19, in contrast to a significant decrease in other age groups. The immune system is activated upon viral infection to combat pathogenic microorganisms [21]. Infants under the age of one, who lack complete immune memory and possess diminished innate and adaptive immunity due to an immature immune system, may be more vulnerable to respiratory viruses, with NPIs potentially exerting limited influence [22]. Nevertheless, neonates acquiring substantial passive antibodies from mothers and older children with more robust immune systems may greatly benefit from the implementation of NPIs as a protective measure against respiratory viral infections.

This study highlights the evident seasonality of certain respiratory viruses, with heightened detection rates for RSV and Flu A during the winter months. Interestingly, during the COVID-19 period, PIV-3 predominantly emerged in autumn and winter, whereas before the pandemic, PIV-3 was primarily detected in summer, followed by spring. As the transmission of flu largely occurs through droplets and contact, the preventive measures undertaken in the early stages of the novel coronavirus outbreak aligned with those employed for flu prevention and control during spring and summer, effectively curtailing flu transmission. Nonetheless, the commencement of the fall semester in September 2020, coinciding with the subsiding domestic COVID-19 situation in China, prompted a return to regular activities in low-risk areas akin to the pre-COVID-19 era. This might account for the spike in PIV-3 infections during the summer of 2020, consistent with pertinent research findings [23].

This study is subject to certain limitations as it encompasses a single-center approach and retrospective analysis, focusing solely on data from children admitted to our hospital. Factors contributing to respiratory virus infection beyond the hospital setting were not explored. In the present study, respiratory virus detection was conducted using an immunofluorescence assay. Indeed, employing real-time PCR could have yielded more accurate results.

In conclusion, RSV infection remains the predominant respiratory tract infection, exhibiting distinct distributions across age groups and seasons. COVID-19 preventive measures effectively contained the transmission of various respiratory viruses. This study comprehensively detected and analyzed respiratory viruses in hospitalized children with acute respiratory tract infections, providing the foothold for the clinical diagnosis and treatment of respiratory tract virus infections during epidemics.

### Electronic supplementary material

Below is the link to the electronic supplementary material.

Supplementary Material 1
