# Supplementary material for: Analysis of respiratory virus detection in hospitalized children with acute respiratory infection during the COVID-19 pandemic
Source: Virol J. 2023 Nov 2;20:253. doi: 10.1186/s12985-023-02218-5 (PMC10623845; doi:10.1186/s12985-023-02218-5)
Supplement: Supplementary file 1 — Supplementary Material 1 [file 12985_2023_2218_MOESM1_ESM.docx]

Supplementary Table S1 the annual percentage of positive respiratory virus detections among hospitalized children with ARIs from 2018 to 2022

|  | **Period before COVID-19n1/n2** (**%**) | | **During COVID-19n1/n2** (**%**) | | | χ^2^ value | P value |
| --- | --- | --- | --- | --- | --- | --- | --- |
|  | 2018 **n1/n2** (**%**) | 2019 **n1/n2** (**%**) | 2020 **n1/n2** (**%**) | 2021 **n1/n2** (**%**) | 2022 **n1/n2** (**%**) |  |  |
| Age |  |  |  |  |  |  |  |
| 0~＜1y | **536/2688(19.94%)** | | **535/2311(23.15%)** | | | **7.605** | **0.006** |
|  | 212/1313 (16.15%) | 324/1375 (23.56%) | 159/631 (25.20%) | 234/864 (27.08%) | 142/816 (17.40%) |  |  |
| 1~＜3y | **173/1223(17.14%)** | | **173/1256(13.77%)** | | | **0.071** | **0.789** |
|  | 62/554 (1.12%) | 111/669 (16.59%) | 51/274 (18.61%) | 64/436 (14.68%) | 58/546 (10.62%) |  |  |
| 3~＜7y | **105/989(10.62%)** | | **99/1036(9.56%)** | | | **0.629** | **0.428** |
|  | 31/444 (6.98%) | 74/545 (13.58%) | 22/170 (12.94%) | 28/353 (7.93%) | 49/513 (9.55%) |  |  |
| 7~＜14y | **7/121(5.79%)** | | **5/158(3.16%)** | | | **1.143** | **0.285** |
|  | 1/45 (2.22%) | 6/76 (7.89%) | 1/17 (5.88%) | 1/28 (3.57%) | 3/113 (2.65%) |  |  |
| 0~＜14y | **821/5021(16.35%)** | | **812/4761(17.06%)** | | | **0.871** | **0.351** |
|  | 306/2356 (12.99%) | 515/2665 (19.32%) | 233/1092 (20.32%) | 327/1681 (19.45%) | 252/1988 (12.68%) |  |  |

n1:number of respiratory virus positive, n2: number of ARIs
